# Inflammatory crosstalk: systemic gut-kidney interplay aggravates tumor host mortality

**DOI:** 10.1038/s44318-025-00457-6

**Published:** 2025-05-22

**Authors:** Héctor Herranz

**Affiliations:** Department of Cellular and Molecular Medicine, University of Copenhagen, Blegdamsvej 3, Copenhagen, 2200N Denmark

**Keywords:** Cancer, Immunology, Microbiology, Virology & Host Pathogen Interaction

## Abstract

Recent work uncovers gut-derived inflammation and renal dysfunction as causally linked drivers of the paraneoplastic syndrome in the fly.

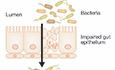

## Teaser

Paraneoplastic syndromes affect multiple organs and are a central cause of mortality in cancer patients. In this issue, Cong et al (2025) report an inflammatory gut-kidney axis mediating systemic complications in *Drosophila* hosts with neoplastic tumors. These results suggest novel potential therapeutic targets in cancer patients.

Paraneoplastic syndromes present complex systemic clinical symptoms in cancer patients, often complicating the clinical management and prognosis of the disease. These syndromes are not directly caused by the tumor itself or its metastases but are initiated by substances produced by the cancer cells or by the immune response triggered by the tumor. In some instances, identifying a paraneoplastic syndrome can indicate the presence of a malignancy (Pelosof and Gerber, 2010). Hence, understanding the specific mechanisms by which these syndromes occur can help in developing diagnostic tests for early detection and targeted therapy.

The immune response triggered by tumor cells is a central driver of paraneoplastic syndromes, causing inflammation and damage in healthy tissues. Because of the tumor-induced immune response, autoantibodies and cytotoxic T cells can target normal cells, leading to various neurological, endocrine, and dermatological symptoms. In addition, the release of cytokines during the immune response can result in widespread inflammation, further exacerbating tissue damage (Graus and Dalmau, 2019; Pelosof and Gerber, 2010). Glomerulopathy is a specific type of paraneoplastic syndrome characterized by glomerular lesions and kidney damage, often caused by the immune response to a tumor. A significant number of cancer patients exhibit glomerular lesions, which are associated with poor prognosis and involve immune complex deposition in the kidneys (Berns and Rosner, 2012; Pani et al, 2016). Understanding these immune-mediated mechanisms is crucial for developing treatments that can modulate the immune response, thereby improving patient outcomes and reducing the severity of these syndromes.

In vivo models can be valuable to understand the complex mechanisms underlying paraneoplastic syndromes and to develop effective treatments, as, in contrast to in vitro studies, they enable the investigation of the complex interactions between cancer cells and distal organs. *Drosophila melanogaster* is an ideal model organism in that respect, due to its genetic tractability and the conservation of many biological pathways with humans (Verheyen, 2022). Besides, the fruit fly relies solely on innate immunity, which is simpler and easier to study compared to the complex adaptive immune system found in mammals. This facilitates the analysis of fundamental immune responses without the interference of adaptive immunity. Notably, *Drosophila* models have been recently used to investigate pathological mechanisms by which cancer cells affect the host organs, leading to paraneoplastic symptoms. For example, recent studies have shown that gut tumors in *Drosophila* can induce kidney dysfunction through specific signaling pathways, providing insights into the mechanisms of tumor-host interaction (Kwok et al, 2024; Xu et al, 2024).

The current work by Cong et al (2025) builds on a recently established model for the transplantation of neoplastic tumors in healthy *Drosophila* hosts (Yang et al, 2019) and reports the appearance of paraneoplastic traits affecting the ovaries, intestines, nephrocytes, fat and muscle tissues, and host metabolism. Consistent with the presence of systemic alterations, flies carrying those tumors exhibited a reduced lifespan, making this model an ideal platform to investigate tumor-host interactions.

**Figure 1:**
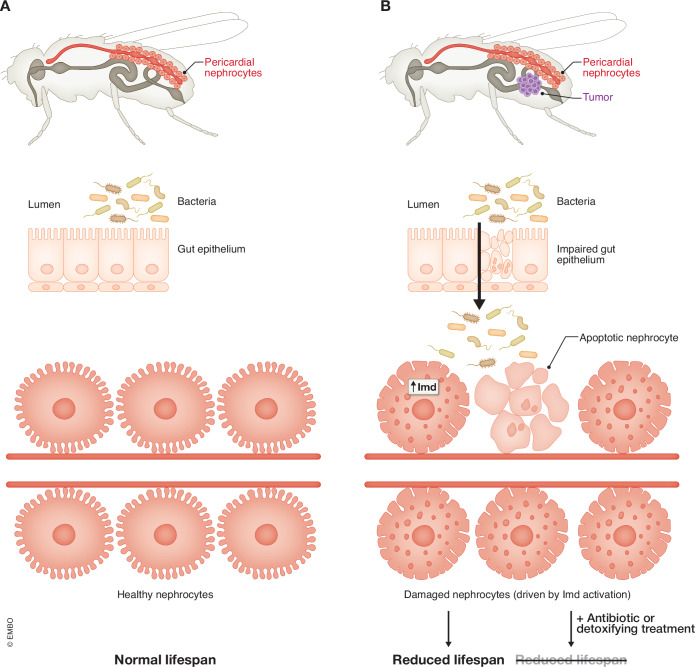
Gut bacterial translocation and immune-activated renal damage define tumor-associated mortality in *Drosophila.* The left column (**A**) illustrates the commensal bacteria, gut epithelium, and pericardial nephrocytes in healthy flies. A fly carrying a neoplastic tumor is shown in the right column (**B**). In this fly, defects in the gut lead to commensal bacterial translocation and consequent activation of the systemic innate immune response via the Imd pathway. This causes damage to host nephrocytes and leads to a reduced lifespan. Treatments reducing the bacterial load (e.g., with antibiotics) ameliorate nephrocyte deterioration and the tumor-associated effect on lifespan.

A detailed analysis of pericardial nephrocytes in tumor-bearing host flies identified defects such as increased vesicle numbers and aberrant lysosomes, disrupted slit diaphragms and basement membranes, and increased apoptosis, which, together, indicate impaired nephrocyte structure and function. The observed defects in pericardial nephrocytes were likely a consequence of compromised gut barrier function in these hosts. RNA-seq and proteomic analyses of tumor hosts showed an upregulation of genes and proteins involved in the Toll and immune deficiency (Imd) signaling pathways. Further investigations revealed a higher bacterial load and dysbiosis in the guts of tumor hosts. Functional analyses revealed that the increased bacterial load triggered Imd/NF-κB activation and subsequent damage in the nephrocytes of tumor-host flies (Fig. 1). These experiments also showed that different bacteria (*Acetobacter* vs. *Lactiplantibacillus*) had varying impacts on nephrocyte damage.

Cong et al (2025) identify the renal system as a central hub of this paraneoplastic syndrome model, wherein the pericardial nephrocytes undergo severe damage due to the elevated immune response triggered by gut dysbiosis and bacterial translocation. Notably, the innate immune response-induced nephrocyte damage is a major contributor to reduced lifespan in the tumor hosts, as the authors (Cong et al, 2025) demonstrate that blocking the Imd/NF-κB pathway in nephrocytes or removing gut bacteria via germ-free derivation or antibiotic treatment ameliorates nephrocyte deterioration and extends the lifespan of tumor hosts (Fig. 1). In addition, treatment with the detoxifying drug AST-120, an oral charcoal adsorbent used in chronic kidney disease patients to remove toxins (Sanaka et al, 1988), also extended the lifespan of the tumor hosts. These findings highlight the critical role of the gut-kidney axis in the paraneoplastic complications observed in cancer-bearing flies, suggesting potential therapeutic targets for mitigating similar complications in cancer patients.

The study highlights the power of *Drosophila* as a model organism to dissect complex biological processes relevant to human diseases. The strength of this study lies in the elegant use of a genetically tractable model to reveal a causal link between tumor-driven changes in the gut of tumor hosts and damage in a distant organ. While further research is needed to fully translate these findings to human cancers, this work provides a compelling framework for understanding the systemic consequences of tumor-host interactions and highlights the gut-kidney axis as a promising area for future investigation and therapeutic development in the context of paraneoplastic syndromes. For example, strategies aimed at maintaining gut barrier integrity, modulating the gut microbiota, or inhibiting the Imd/NF-κB pathway in the kidney could be explored. Moreover, the results showing that AST-120 improved nephrocyte morphology and prolonged lifespan in tumor-bearing flies further support the potential of targeting bacterial metabolites in cancer patients.

The findings presented by Cong et al (2025) suggest that monitoring gut health and markers of immune activation and renal function could be important for early detection of paraneoplastic complications and potentially for predicting prognosis in cancer patients. Implications for personalized medicine approaches can also be inferred from this article. The results showing that different bacteria had varying impacts on nephrocyte damage suggest that the specific composition of the gut microbiota might influence the susceptibility and severity of paraneoplastic syndromes. This could open avenues for exploring personalized medicine approaches based on individual patient microbiome profiles.

In conclusion, the results presented by Cong et al (2025) provide a compelling link between tumor-induced gut dysbiosis, bacterial translocation, and immune-mediated renal damage in *Drosophila*, offering novel insights into the pathogenesis of paraneoplastic syndromes and highlighting promising avenues for future biomedical research and the development of therapeutic strategies to improve the health outcomes of cancer patients.
